# The Impact of a Community Partner Research Training Program

**DOI:** 10.1007/s13187-025-02718-8

**Published:** 2025-08-22

**Authors:** Oluwademilade Adefarati, Rickisa Yearwood, Yawei Song, Amy E. Leader, Karen Glanz, Evelyn T. Gonzalez, Scott W. Keith, Nilsa Graciani, Thierry Fortune, Charnita M. Zeigler-Johnson

**Affiliations:** 1 Cancer Prevention and Control, Fox Chase Cancer Center, Philadelphia, PA USA; 2 Office of Community Outreach and Engagement, Sidney Kimmel Cancer Center, Philadelphia, PA USA; 3 Perelman School of Medicine and School of Nursing, University of Pennsylvania, Philadelphia, PA USA; 4 Community Outreach and Engagement, Fox Chase Cancer Center, Philadelphia, PA USA; 5 Division of Biostatistics and Bioinformatics, Sidney Kimmel Medical College, Thomas Jefferson University, Philadelphia, PA USA; 6 STEM and Medical Assisting, Esperanza College of Eastern University, Philadelphia, PA USA; 7 Department of Public Health, City of Philadelphia, Philadelphia, PA USA

**Keywords:** Cancer disparities, Cancer research, Training program, Community-engaged research

## Abstract

As part of its mission to address disparities in cancer outcomes and research participation, the Philadelphia Communities Conquering Cancer (PC3) coalition conducted a Community Partner Research Training program. The goal was to increase knowledge about cancer research and to prepare community members to partner on research activities. Eighteen out of 36 applicants were selected for a 5-week training program on research methods. Trainees completed baseline (*N* = 18) and follow-up surveys (*N* = 10) to measure knowledge acquisition. Demographic data were analyzed with descriptive statistics. For repeated cross-sectional group comparisons, Wilcoxon nonparametric tests were used to test statistical differences between medians on baseline and follow-up surveys. *P*-values ≤ 0.05 were considered statistically significant. Trainees were mostly female (78%). Median age was 61.5 years. Forty-four percent were Black, 22% White, 17% Asian, and 17% other race. Twenty-eight percent were Hispanic. Forty-four percent were cancer survivors. We observed that perceived knowledge about research generally increased post-training from a median of 2 to 4 across a 5-point Likert scale (*p* < 0.001). This increase is shown for several items, including “how to conduct a research study” and “how to share findings with the community.” Program ratings averaged 4 out of 5, indicating positive participant experiences in program organization and content. Involving diverse populations in cancer research is crucial for reducing health disparities. This training program demonstrated that it is feasible to increase knowledge about cancer research methodology among community partners. These results will be used to guide future research activities with PC3.

## Introduction

### Background

Philadelphia, a diverse city in Pennsylvania, faces significant cancer disparities, particularly among marginalized populations [1]. Cancer is a leading cause of death, with risk factors like obesity, smoking, and environmental exposures being more prevalent in Philadelphia compared to national averages [2]. Racial and ethnic minority populations, often residing in medically underserved areas, may also belong to socially vulnerable groups that experience low health literacy and face barriers to accessing cancer care [1, 3].

Community partnerships are a core piece in community-based research, strengthening the quality of translational research for health equity progression and are vital for enhancing research engagement and trust. They empower communities by providing transparency about research processes, promoting trust, and encouraging active research participation [4]. Connecting with community members through partnerships, specifically key stakeholders, can also increase the potential for these communities to improve their research capacity, which indicates the ability to understand, engage with, and conduct research [5]. This knowledge development is reciprocal as community stakeholders provide their prior knowledge and experiences to better address meaningful research questions [6]. With this knowledge, researchers can be more responsive to community needs, ensure culturally relevant interventions, and disseminate findings that are easily accessible to the public [7].

Advancements in community research have led to the development of community research training programs. These programs benefit science by providing community collaborators with training in community engagement processes, which can improve the probability of diverse recruitment and enhance the applicability of study results [7, 8]. They help reduce health disparities by increasing awareness about health issues [5, 9]. Under the direction of community and cancer center leaders, these programs contribute to building strong relationships with underrepresented and marginalized populations, which can help increase trust and participation in future research studies and clinical trials [10, 11].

Addressing these challenges requires a multidisciplinary approach, including community engagement, transparent communication, and tailored strategies to make research more inclusive and accessible [9]. Community engagement is essential for promoting a better understanding of research goals, processes, and the importance of including diverse trainees among historically marginalized communities [9, 12]. Diverse participation in research, including clinical trials, is crucial for promoting health equity and ensuring the translation of study results to various populations.

### Philadelphia Communities Conquering Cancer (PC3)

The Philadelphia Communities Conquering Cancer (PC3) coalition was established in 2022, bringing together community stakeholders and the three NCI-designated cancer centers in Philadelphia: Fox Chase Cancer Center at Temple University, Abramson Cancer Center at the University of Pennsylvania, and Sidney Kimmel Cancer Center at Thomas Jefferson University [13]. PC3 aims to reduce health disparities through community engagement, information sharing, and collaborative research. Creating a united front with all three NCI-designated cancer centers in Philadelphia promotes an “all hands-on deck” approach to community-based cancer education research [13].

As suggested by previous research [5, 10], involving community members in various aspects of research (e.g., study design, recruitment, informed consent) can enhance engagement, reduce mistrust, and support cancer-related initiatives. Specifically, the PC3 program aimed to equip the Philadelphia community with research skills to actively engage in cancer-related activities, addressing disparities in cancer care and outcomes [13]. This training program also introduces a hybrid approach, combining in-person and virtual components, which allows for broader accessibility and adaptability post-2020. Here, we detail the recruitment process, the curriculum’s elements, and evaluation strategies that distinguish this program as an innovative step forward in community-engaged cancer research.

## Methods

### PC3 Training Program

The PC3 Community Partner Research Training (CPRT) program is centered around training community members in the principles of patient-centered outcomes research (PCOR), community-engaged research (CER), cancer disparities, and health equity. The program used Research Fundamentals Modules from the Patient-Centered Outcomes Research Institute (2024) and PC3-developed clinical research training sessions.

### Recruitment

PC3 used existing community and patient connections for recruitment. Flyers, distributed in person or by email, and word-of-mouth were used to advertise the free PC3 CPRT program for community members. Interested applicants (*N* = 36) were scheduled for interviews with PC3 team members. Interviews were conducted using a Participant Interview Guide (Appendix) created by the program leaders. Each interview had at least two interviewers who used the guide to rate candidates. Scores were averaged, and candidates were ranked. Selected candidates with the highest scores were determined through consensus across interviewers and program leaders. These candidates (*N* = 18) were notified by email. There were no differences in mean age, race/ethnicity, or educational attainment of those who were selected compared to those who did not participate in the program. Onboarding included candidates receiving the training program schedule and documentation for processing stipends. The stipends helped cover travel expenses and compensated community members for their time.

### Training Program Content

The training program consisted of five sessions, each held once per week (Appendix). The first training session outlined the PC3 program by introducing team members, sharing Philadelphia cancer disparity statistics, and explaining the role PC3 Community Partners would take as future contributors to the research infrastructure of PC3. Trainees also received information about the PCORI Research Fundamentals Training and the University of Pennsylvania Community Partner Training Registration [14]. The UPenn Training is a lay-friendly human subjects training (similar to Institutional Review Boards’ requirements) and offers a free 2-h online course for community members interested in research. It covers the basics of research principles and explains the duties of community researchers with four modules (1. Introduction to Research; 2. Human Research: Rules and Regulations; 3. Recruitment and Informed Consent; 4. Being Careful with Research Information).

Throughout the training sessions, trainees were provided with resources, including PowerPoint presentations, Patient-Centered Outcomes Research Institute (PCORI) Research Fundamentals Modules [15], and activities related to the weekly research topics. Fundamentals training included developing research questions, recruiting trainees, and sharing research findings. Each session included open discussion and interactive activities. After program completion, individuals received a PC3 participation certificate.

### Data Collection

Trainees completed the PC3 CPRT Baseline Survey. The Baseline Survey included a set of statements with a 5-point Likert scale regarding the participant’s familiarity/perceived knowledge of the subject matter related to conducting research (1 = not at All, 2 = a little, 3 = somewhat, 4 = a great deal, and 5 = completely). The survey also included demographic and open-ended questions on research experience and connections to cancer. This survey was distributed during the first in-person session.

The PC3 CPRT follow-up survey was completed during the final session. The follow-up survey had the same thirteen 5-point Likert-scale statements about perceived knowledge of the subject matter related to conducting research. It also included statements regarding how trainees felt about program content. Open-ended questions asked trainees to provide qualitative information regarding cancer topics of interest and recommendations to improve the PC3 CPRT program. In addition, we asked trainees which PCORI online modules they had watched (1 = none of it, 2 = part of it, and 3 = all of it).” Both baseline and follow-up surveys were de-identified to help trainees feel comfortable answering background questions truthfully.

### Analytic Plan

Frequency counts and percentages were calculated to describe baseline demographics of the sample. Medians, means, and other summary statistics were computed to summarize study outcomes data and compare the baseline and follow-up survey results. Wilcoxon rank-sum tests were conducted to evaluate differences in the distributions of Likert-scaled responses to the 13 training questions at baseline and follow-up surveys, using a nominal *α* = 0.05 to judge the significance of the* p*-values. All data analyses were conducted with SPSS v29.0 (IBM, Armonk, NY) and SAS v9.4 (SAS Institute, Cary, NC).

Researchers categorized open-ended comments based on common themes for qualitative data. Themes were derived from the participant responses (e.g., Online Learning & Community Engagement) to determine prevalent concerns and/or findings. Categorization followed an iterative process among PC3 researchers. Qualitative data were compared in tables.

## Results

Survey data showed that program trainees (*N* = 18) were diverse and came from varying age groups, racial/ethnic backgrounds, levels of educational attainment, and professional affiliations (see Table 1). Fourteen (78%) of the trainees were female. Ages ranged from 21 to 76 years (median = 61.5 years). Eight (44%) were Black, four (22%) White, three (17%) Asian, and three (17%) other. Five (28%) were Hispanic or Latino. Eleven (61%) were college graduates, and eight (44%) were cancer survivors.

**Table 1:** PC3 CPRT program trainee demographics (*n* = 18)

	*N*	Percent
Gender		
Male	4	22%
Female	14	78%
Race		
Asian	3	17%
Black	8	44%
White	4	22%
Other	3	17%
Ethnicity		
Hispanic or Latino	5	28%
Not Hispanic or Latino	13	72%
Age		
18–25	3	17%
26–40	2	11%
41–55	3	17%
56–70	6	33%
70+	4	22%
Patient status		
Cancer survivor	8	44%
Education		
HS or GED	1	5%
Tech/Trade	1	6%
Some college, Associates	5	28%
College Graduate	7	39%
Postgraduate	4	22%
Employment		
Working full time	6	33%
Working part time	3	17%
Student or Caregiver	1	11%
Retired	6	33%
Other	2	17%

Table 2 shows summaries of the Likert-scale data from the baseline and follow-up surveys. There were statistically significant differences between the response distributions for the 13 questions. The median scores were higher at follow-up than baseline, increasing on average from 2.0 to 4.0, indicating gained knowledge among trainees.

**Table 2:** PC3 CRPT baseline and follow-up survey response summary (*N* = 18)

Item	Pre			Post			Sig
Question	Median	Mean	SD	Median	Mean	SD	*P*-value*
1. Current cancer disparities in Philadelphia	2	2.11	0.81	3.5	3.6	0.66	**0.002**
2. The major risk factors for cancer	3	3.06	0.97	4	3.64	0.48	**< 0.001**
3. The social determinants of health	3	2.71	0.96	4	3.64	0.72	**< 0.001**
4. The steps involved in conducting a research study	2	2.41	1.03	4	3.57	0.90	**< 0.001**
5. How to recruit people to participate in research	2	2.41	0.91	4	3.71	0.88	**< 0.001**
6. How to collect research data	2	2.44	1.12	4	3.64	1.11	**< 0.001**
7. The different types of research (qualitative and quantitative)	2	2.13	1.05	4	3.79	0.86	**< 0.001**
8. How to share findings from research back to the community or trainees	2	2.33	0.94	4	3.57	0.50	**< 0.001**
9. Why cancer clinical trials are important	4	3.41	1.09	4	4.43	0.50	**< 0.001**
10. What an IRB does related to the ethical approval of research	2	2	1.24	3.5	3.57	0.82	**< 0.001**
11. The purpose of informed consent in research	3	3.28	1.15	4	4.29	0.59	**< 0.001**
12. Ways that community members can be involved in cancer research	3	2.67	1.11	4	3.71	0.59	**< 0.001**
13. The value of including community members in cancer research	3	3.39	1.06	4	4.21	0.67	**< 0.001**

*Computed from Wilcoxon Test for medians

A summary of the Likert-scaled responses to the five program rating questions regarding organization, content, in-person training sessions, Zoom (online conferencing platform) training sessions, and online human subjects training program (PCORI) is shown in Table 3. Ratings averaged over 4.5 out of 5 for all categories, indicating positive participant experiences (Table 3).

**Table 3:** PC3 CPRT follow-up survey program ratings summary (*n* = 12)

Areas	Mean (SD)
1. Organization	4.64 (0.64)
2. Content	4.73 (0.62)
3. In-person training	4.64 (0.64)
4. Zoom training	4.45 (0.66)
5. Online human subjects training	4.55 (0.66)

Scores are from 1 to 5, with 5 being the highest

Responses to open-ended questions in the follow-up survey revealed that trainees had various interests in learning different cancer topics, including genetic factors and the impact of long-term chemotherapy. Recommendations included:

1. Extending the training program—Several trainees recommended extending the training program beyond five weeks, with additional breaks to facilitate better assimilation and application of the material. One participant noted, “The training class should be a couple of weeks longer in order to gain a better understanding of all the terms.”
2. Offering more in-person sessions—The majority of the program was conducted over Zoom in a “lecture-based” format. Trainees expressed a preference for more in-person sessions to enhance engagement and learning. The virtual format also contributed to “Zoom fatigue,” defined as “a feeling of exhaustion from participating in video conference calls,” [16] which further challenged trainees’ ability to fully absorb the material. One participant commented, “The training class should be a couple of weeks longer in order to gain a better understanding of the terms.” Another stated, “Information overload on Zoom.” A third shared, “More training in person, being on Zoom all day for work makes training hard.”
3. Engaging in more group discussions and dialogue—Several trainees brought professional experiences in healthcare and community organizing to the program. As a result, they suggested the research fundamentals sessions could be condensed into refresher courses in future training sessions, perhaps leveraging the experiences of individuals in the group and tailoring the program’s content to the specific needs of the trainees.
4. Incorporating more hands-on community training experiences—Trainees emphasized the importance of integrating more hands-on training experiences in community settings to enhance learning. Specific suggestions included going into local community spaces like churches or grocery stores to explore community needs and discuss the needs and approaches tailored to those environments. One participant stated, “Take programs into churches and community centers.”

Future iterations of the program will consider these recommendations to improve training outcomes.

## Discussion

Implementing the training program for community partner researchers required collaboration and leveraging existing resources among local cancer centers and community partners. Over the program’s duration, PC3 community partners’ understanding and awareness of general cancer topics improved, within and outside of research. PC3 CPRT trainees showed statistically significant improvements in knowledge about cancer risk factors, disparities, and conducting cancer research. Compared to similar community-based participatory research (CBPR) training programs, PC3 is a newer training, capitalizing on PCORI resources and using an online Zoom platform, which gained popularity during the COVID-19 pandemic. PC3’s structure is not entirely unique to the participating institutions but is primarily based on existing tools and metrics. Thus, individuals can easily access many of the PC3 training resources utilized, making this research knowledge accessible to community members.

Similar CBPR and partnership training programs have been created in other settings across disease types within the past 10 years. A 2018 CBPR from Northwestern University’s Center for Health Equity Transformation created a community scientist panel to support the work of cancer research teams studying adverse environmental exposures, called the Metals Project [17]. The Meharry Vanderbilt Community Engaged Research Core-Community Research Training Program (MVC-CRT) is another example similar to PC3’s framework. MVC-CRT was piloted in 2016 and followed a “six-session community-based organization (CBO) and three-session community member (CM) curricula” based on stakeholder goals across public health research topics [18]. The Boston University Clinical & Translational Science Institute (BU-CTSI) also started a “Connecting Community to Research” pilot program for Boston communities to partner with BU to advance the “health outcomes” [19]. The training was interactive and focused on CER principles, with sessions offering opportunities to engage with local study teams on research [19]. Also, the Patient and Investigator Voices Organizing Together (PIVOT) Program at the University of Kansas Cancer Center highlights collaboration between investigators and community stakeholders with relevant lived experiences, centering the patient perspective to enrich research efforts [20]. PIVOT’s focus on increasing patient engagement, through stakeholder leadership, to improve healthcare outcomes has led to an impactful program that enriched the quality of the center’s research outcomes [20].

Public health research has advanced CBPR practices, particularly for diseases and health outcomes that disproportionately impact marginalized populations. Black communities, in particular, frequently encounter less information and empathy from healthcare providers [21]. Many diverse communities have endured neglect, abuse, and exploitation in medical research, causing skepticism. These long-lasting effects are compounded by current healthcare disparities, emphasizing the need for systemic change and novel community-based interventions [12]. Impacting health equity requires understanding disparities and working directly with affected communities. Philadelphia’s diverse demographic includes Black (39.5%), White (34%), Hispanic (15.8%), and Asian (8.2%), with over a quarter of residents being immigrants or from immigrant families [22]. These communities experience higher cancer incidence and mortality rates compared to non-minority groups, driven by limited healthcare access, lack of health insurance, and cultural barriers [3, 23].

Although trainees identified benefits of this program, they also shared that virtual and lecture-based training sessions limited opportunities for engagement, interaction, and hands-on learning. While virtual training provides flexibility, it also introduces the risk of Zoom fatigue [16]. Trainees shared that they struggled to remain focused throughout virtual sessions. Additionally, trainees discussed wanting a longer training program to better understand the educational material and to feel more confident going out into their communities with their gained knowledge.

The PC3 CPRT Program brought together a diverse group of trainees, reflecting the diversity of Philadelphia’s communities. In addition to learning about barriers to CER, trainees were taught the process of informed consent, data collection, and dissemination of research findings. Furthermore, the training staff, including individuals from all three centers and the community advisory board co-chairs, with various professional backgrounds (social work, nursing, epidemiology, health advocacy), provided diverse guidance and insights to trainees. These resources will be available to trainees as they continue in various roles supporting research infrastructure in the cancer centers. Six trainees (33%) have already accepted opportunities to support research as IRB and grant reviewers. Others are considering applying for PC3 funding to co-lead community-driven cancer research studies.

### Limitations

The PC3 training program survey structure restricted the analytic processes. Deidentified data was collected without assigned record IDs; therefore, the Program’s baseline and follow-up surveys could not be matched by participant. Therefore, results could not demonstrate individual knowledge acquisition or changes by demographic variables.

## Conclusion

Through conducting the PC3 CPRT Program, PC3 institutions and researchers gained a collection of useful knowledge. Developing a team of diverse community partners to be PC3 Community Researchers will help to address health inequities in cancer research. The next steps include linking the Community Researchers with research projects at the cancer centers to facilitate community-engaged cancer research and help sustain the PC3 program’s relevance and growth in the years to come.

## Appendix

### PC3 Community Research Partner Training Program Participant Interview Guide

The interviewer explains the PC3 community research partner training:

- Philadelphia Communities Conquering Cancer (PC3) is a city-wide coalition formed in 2022. The coalition is led by three NCI-designated cancer centers in Philadelphia: Abramson Cancer Center at UPenn, Sidney Kimmel Cancer Center at Thomas Jefferson University, and Fox Chase.
- The coalition’s mission is to listen, engage, and empower communities, organizations, and individuals to reduce cancer disparities. PC3 does this by aligning resources, sharing information, inspiring research, and reducing barriers to cancer prevention, early detection, and treatment for all Philadelphians.
- The PC3 Community Research Partner Training program aims to educate and inform community members about the research process and how they can be involved in new and ongoing cancer-related research in the Philadelphia community.

**Interview Questions for Community Research Partners**

Interviewee:

Background: How did you hear about the PC3 community research partner training program?

- What is the main reason you are interested in this training?
  ○
- What do you hope to gain from participating in the PC3 Community Research Partner Training program?
  ○
- Do you have a background in science or healthcare?
  ○
- What does “community research” mean to you?
  ○
- Are you comfortable with public speaking?
  ○
- Are you comfortable using Zoom?
  ○
- Do you live or work in Philadelphia? What are your commitments to the Philadelphia community? (We are really looking for the commitment piece)
  ○
- If community members asked, “What are the next steps or expectations from the people who completed the training?”
  ○
- Do you have other questions for us? General meeting attendance questions.

Interviewer ratings:

**Table:**

Item (scored from 1 [worst] to 5 [best])	Interviewer 1	Interviewer 2
On time for the meeting		
Communication skills		
Maturity, professional and respectful attitude		
Dependability and a strong work ethic		
Adaptability and flexibility		
Good/agreeable personality		
Interest in research and motivation level		
Understanding of the program expectations and role		
Total		40

If both interviewers are a definite “yes” at the interview:

- Follow-up and disclose the research element, compensation ($250 upon the time of completion of all the training sessions), etc., and gather verbal consent and the W-9 form.

PCORI Community Research Partner Training Curriculum

**Table:**

Session	Topics	PCORI research fundamentals training modules
**1** 4/2	**Cancer disparities in Philadelphia** **Introduction to PC3 and Prioritized Research Agenda**	**The PCORI Approach, Engaging in Research** https://www.pcori.org/engagement/research-fundamentals/engaging-stakeholder-driven-research
**2** 4/9	**Addressing Disparities and Partnering with Vulnerable Communities** **Introduction to Research Fundamentals**	**Module 1. Developing Research Questions** https://www.pcori.org/engagement/research-fundamentals/developing-research-questions **Module 2. Designing the Research Study** https://www.pcori.org/engagement/research-fundamentals/designing-research-study
**3** 4/16	**Introduction to Informed Consent and overview of Community Partner (CITI) training** **Introduction to Data Collection**	**Module 3. Planning Patient-Centered Consent and Study Protocols** https://www.pcori.org/engagement/research-fundamentals/planning-patient-centered-consent-study-protocols
**4** 4/23	**Panel Discussion—Community Engaged Research** **Introduction to Research Outreach and Recruitment**	**Module 4. Sampling, Recruiting, and Retaining Study Participants** https://www.pcori.org/engagement/research-fundamentals/sampling-recruiting-retaining-study-participants
**5** 5/4	**Introduction to Results Dissemination**	**Module 5. Understanding and Sharing Research Findings** https://www.pcori.org/engagement/research-fundamentals/understanding-sharing-research-findings
